# Triple-Negative Breast Cancer: Intact Mismatch Repair and Partial Co-Expression of PD-L1 and LAG-3

**DOI:** 10.3389/fimmu.2021.561793

**Published:** 2021-02-24

**Authors:** Shafei Wu, Xiaohua Shi, Jing Wang, Xuefei Wang, Yuanyuan Liu, Yufeng Luo, Feng Mao, Xuan Zeng

**Affiliations:** ^1^ Department of Pathology, Peking Union Medical College Hospital, Molecular Pathology Research Center, Chinese Academy of Medical Sciences, Beijing, China; ^2^ Department of Breast Surgery, Peking Union Medical College Hospital, Chinese Academy of Medical Sciences, Beijing, China

**Keywords:** CD8, LAG-3, PD-L1, microsatellite instability, triple-negative breast cancer

## Abstract

**Background and Aim:**

Poor response to immune checkpoint inhibitors (ICIs) has been observed in most triple-negative breast cancer (TNBC) cases (around 80%). Our aim was to investigate the status of mismatch repair (MMR), microsatellite instability (MSI), programmed death-ligand 1 (PD-L1), and lymphocyte-activation gene 3 (LAG-3) in TNBC.

**Methods:**

A total of 74 TNBC samples were retrospectively analyzed. MMR and MSI were evaluated by immunohistochemistry (IHC) and polymerase chain reaction (PCR) using Promega 1.2 and NCI panels, respectively. PD-L1, LAG-3, and CD8 expression was assessed by IHC.

**Results:**

None of the cases demonstrated deficient MMR (dMMR) or MSI. In total, 43/74 cases (58.1%) were PD-L1+, including 1 tumor PD-L1+, 25 tumor-infiltrating lymphocytes (TILs) PD-L1+, and 17 cases involving concurrence of tumor and TIL PD-L1+. The rate of TIL PD-L1+ was remarkably higher than that of tumor PD-L1+ (P<0.001). We identified 20 LAG-3+ cases (27.0%, 20/74), all of which were PD-L1+. Co-expression of PD-L1 and LAG-3 was noted in 46.5% (20/43) of the PD-L1+ population. In the LAG-3+ subtype (co-expression of PD-L1 and LAG-3), high correlation between TILs PD-L1+ and LAG-3+ was observed (P<0.01). A high frequency of CD8+ (98.6%, 73/74) was observed.

**Conclusion:**

dMMR/MSI characteristics may not be a practical predictive marker for ICIs in TNBC. PD-L1+ is more common in TILs than in tumors. In the PD-L1+ population, approximately half of the cases showed LAG-3 co-expression. For patients with a poor response to PD-1(L1) mono ICI, dual blockade of PD-1(L1) and LAG-3 may be a viable option for the management of TNBC.

## Introduction

Breast cancer (BC) is a heterogeneous disease. Molecular types, essentially including luminal, human epidermal growth factor receptor 2 positive (HER2+), and triple-negative, for which clinical outcomes are closely tied to the corresponding treatment, are categorized based on the status of estrogen receptor (ER), progesterone receptor (PR), and human epidermal growth factor receptor 2 (HER2). Unlike luminal (hormone receptor-positive) and HER2+ (HER2-rich) patients, who benefit from endocrine therapy and HER2-targeted therapy, respectively, cytotoxic chemotherapy is the standard strategy for the advanced triple-negative (HER2-, ER-, and PR-) cases, which account for 15%–20% of invasive BCs. The exception is a small number of TNBC cases with *BRCA* gene mutation (approximated 11-25%) that respond well to poly (ADP-ribose) polymerase (PARP) inhibitors. In general, the prognosis of triple-negative breast cancer (TNBC) is relatively poor, and the tumors recur rapidly (1–3).

Programmed death-ligand 1 (PD-L1), which is a negative regulator of T-cell activation, is expressed in many cancers. The interaction of programmed cell death 1 (PD-1) and its ligand, PD-L1, is known to act as a critical blockade pathway in malignant tumors for regulating immune escape. Therefore, exploring the mechanism of immune regulation involving the PD-1/PD-L1 axis, innovating blocking drugs, and implementing the related clinical practice has attracted a lot of attention among researchers. Naturally, inhibitors of PD-1(L1) are expected to be promising options for the treatment of TNBC (4, 5).

In the last two years, promising findings about the therapeutic effects of anti-PD-1(L1) agents in TNBC have been published. For example, the efficacy in patients who received atezolizumab (a monoclonal antibody targeting PD-L1) plus chemotherapy was significantly better than in those treated with chemotherapy alone. Moreover, PD-L1+ patients had prolonged median overall survival in advanced TNBC (6). Therefore, PD-L1 expression detected by immunohistochemistry (IHC) was considered as one of the most essential predictors for identifying potential beneficiaries of PD-1(L1) checkpoint inhibitors, and these inhibitors were approved by the US Food and Drug Administration (FDA) (https://www.fda.gov/medical-devices/vitro-diagnostics/list-cleared-or-approved-companion-diagnostic-devices-vitro-and-imaging-tools/).

However, clinical response to PD-1(L1) blockers as a single-drug therapy was quite limited, and sufficient benefit has not yet been achieved in the majority of TNBC patients based on the published data.

For example, in a phase I study of 116 patients with metastatic TNBC (mTNBC) to whom atezolizumab was administered, the objective response rates (ORRs) were 24% and 6% in first-line and second-line or greater for patients, respectively, and the ORRs were 12% and 0% for the PD-L1≥1% and <1% subgroups, respectively (7). Likewise, in a phase II study, KEYNOTE-086, 84 cases of PD-L1+ mTNBC were enrolled in first-line therapy with pembrolizumab (a PD-1 inhibitor). The ORR was 21.4% (8). Most TNBC cases had no benefit from anti-PD-1(L1) agents. Therefore, besides PD-L1 expression, it is important to investigate additional biomarker(s) to evaluate the efficacy of immune checkpoint inhibitors (ICIs) such as anti-PD-1(L1) and to determine which biomarker(s) may serve as indicator(s) for the combination regimens (e.g. ICI plus ICI) other than ICI plus chemotherapy.

Solid tumors with impaired DNA mismatch repair (MMR) system {mainly including MLH1, PMS2, MSH2, and MSH6 molecules from which phenotype microsatellite instability (MSI) was determined} responded well to ICI therapy (e.g. pembrolizumab) due to the existence of mutation-related neoantigens presumably derived from high tumor mutation burden, which was recognized by the immune system and triggered T-cell function upregulation. High concordance between high-frequency microsatellite instability (MSI-H) and deficient mismatch repair (dMMR) was revealed in colorectal cancer in many investigations (9–11). Nevertheless, the available results about MMR (conventionally detected by IHC) or MSI {usually detected by polymerase chain reaction (PCR)} status in TNBC are still limited and contradictory to the data compared to colorectal and endometrial carcinoma (started with Lynch syndrome research) for which there were relevant guidelines for MMR and MSI detection. Although the frequency of dMMR and/or MSI tumors in TNBC is very rare (0.04-1.8%), according to some investigators, as much as 20.5% of homogeneous dMMR and 9.1% of heterogeneous dMMR, 90% of which were microsatellite stable (MSS) and showed highly discordant results between IHC and PCR, have also been reported (12–14). Faced with the current situation in which tumors with dMMR/MSI-H obtained durable immune responses from ICIs which were approved by FDA but had insufficient and contrary findings about the molecular features, it is necessary to conduct more studies on this pathway for searching other biomarkers that can help identify patients who may potentially benefit from these treatments (15).

With respect to investigating the biomarker(s) to assess the efficacy of ICI *via* PD-1 (L1) blockade, a new checkpoint, lymphocyte-activation gene 3 (LAG-3), which is an inhibitory receptor expressed on activated T lymphocytes and down-regulates T cell-mediated immune response *via* LAG-3/MHC class II (ligand of LAG-3) interaction, has been the focus of recent research. Upregulated LAG-3 expression has been observed in some malignant diseases. Effector T lymphocytes were energized by blocking LAG-3 based on previous investigations. In addition, co-expression of PD-L1 and LAG-3 was identified in approximately 50% of PD-L1+ cases that were estrogen receptor-negative (16). Therefore, LAG-3-mediated immunosuppression was exhibited depending on the biological behavior of LAG-3 exposure. It is inferred to be a potential prospect for interdicting LAG-3 and exploring the combination of anti-PD-1(L1) and anti-LAG-3 strategies. From the available data, the responsiveness to PD-1(L1) inhibitor was improved when the dual inhibition immunotherapeutic strategy, anti-PD-1(L1) plus anti-LAG-3, was applied (17, 18). Furthermore, trials focusing on the evaluation of clinical response to LAG-3 suppressor (IMP321, a recombinant soluble LAG-3Ig fusion protein) plus chemotherapy (paclitaxel) in BCs (e.g., NCT00349934) as well as IMP321 plus pembrolizumab in advanced solid tumors (e.g., NCT2676869), were carried out, respectively (19). Based on the findings described, examination of the LAG-3 expression and co-expression of PD-L1 as well as elucidation of the tumor microenvironment referring to immunotherapeutic resistance to anti-PD-1(L1) were all extremely valuable for adopting suitable immunologic treatment and improving the clinical effect of anti-PD-1(L1) therapy in TNBC.

Additionally, presence of cytotoxic CD8+ T cells has been found to indicate a favorable prognosis. High-frequency expressions of PD-L1 and tumor infiltrating lymphocytes (TILs) were distinguished, and CD8+ TILs attracted further attention in TNBC, although very few related studies have been conducted (12, 20). Consequently, the meaningful association between CD8+ TILs and the predictive markers of response to ICIs need to be assessed in combination and stratified precisely.

Our purpose was to evaluate the status of MMR/MSI, PD-L1, LAG-3+ TILs, and CD8+TILs and to survey the relationship between these markers in TNBC.

## Materials and Methods

### Patients and Specimens

A total of 74 formalin-fixed paraffin-embedded specimens from primary and metastatic triple-negative invasive breast cancers, including 62 invasive breast cancers of no specific type cases and 12 invasive lobular carcinoma, archived in Peking Union Medical College Hospital between December 2015 and December 2018 were enrolled in the study. The ER, PR, and HER2 status were identified using protein expression and gene amplification by IHC (ER, PR, and HER2) and fluorescent *in situ* hybridization (FISH, reflex HER2 testing for IHC equivocal samples) assays along with the conventional histopathological diagnosis. The clinicopathological characteristics of the patients are listed in **Table 1**. This retrospective study was approved by the Institutional Review Board of Peking Union Medical College Hospital and was performed in accordance with the Declaration of Helsinki and the ethical standards for medical research involving human participants.

**Table 1:** The clinicopathologic characteristics of 74 patients with TNBC.

Characteristics	Number of patients	Percent (%)
Gender		
Female	74	100.0
Male	0	0.0
Age		
<50	30	40.5
≥50,<60	21	28.4
≥60,<70	15	20.3
≥70	8	10.8
Degree of tumor differentiation		
High	3	4.1
Middle	32	43.2
low	39	52.7
Distant metastases		
0	52	70.3
1	22	29.7
Tumor size		
≤2cm	43	58.1
≤5cm,>2cm	25	33.8
>5cm	6	8.1

### MMR Protein Expression Detection by IHC

IHC staining was conducted to assess the expression of four MMR proteins, MLH1, MSH2, MSH6, and PMS2 on 4 μm formalin-fixed paraffin-embedded slides. According to the manufacturer’s protocols, primary monoclonal antibodies against MLH1 (clone M1), MSH2 (clone G219-1129), MSH6 (clone SP93), and PMS2 (clone A16-4) were used based on Ventana BenchMark autostainer (Ventana Medical System, Inc., Tucson, AZ, USA). dMMR was considered when any of the four MMR proteins were completely absent in the nuclear staining of tumor tissue, while concurrent positive benign cells were found in adjacent tissues, and intact IHC staining of these four antibodies was classified as proficient MMR (pMMR) according to the interpretation criteria described previously (21). For the pMMR subtype, MLH1, PMS2, MSH2, or MSH6 protein was scored as high if IHC staining was found in more than 50% of tumor cells according to a previous study (22).

### Intrinsic Subtype Stratification by IHC and FISH

The expression of ER, PR, and HER2 proteins was evaluated on 4μm thick tissue sections by IHC using Ventana BenchMark automated immune stainer with antibodies of SP1, IE2, and 4B5 clones (Ventana Medical System, Inc., Tucson, AZ, USA), respectively, according to the manufacturer’ s instructions. The tumors were classified as positive for ER or PR if immunoreactivity was found in ≥1% of tumor cell nuclei, according to ASCO/CAP recommendations for immunohistochemical testing of ER and PR in BC (23). HER2 status was detected by IHC in the initial examination, followed by FISH testing for IHC equivocal cases. FISH was performed on 4 μm sections using the Thermo-Brite Elite automated FISH slide prep system (Leica, Richmond, CA, USA) with a PathVysion HER2 DNA probe kit (Vysis/Abbott, Abbott Park, Illinois) as the standard protocol. HER2 IHC and FISH slides were scored according to the ASCO/CAP HER2 testing guidelines: IHC 0, 1+, 2+, and 3+ were determined. IHC 0 and IHC 1+ were classified as HER2-negative, and IHC 3+ was classified as HER2-positive. IHC 2+ was considered as HER2 equivocal and was further confirmed by FISH assay. HER2 FISH positivity was determined when the ratio of HER2/CEP17 ≥2.0 or the average HER2 signal/tumor cell ≥6.0, with a ratio of HER2/CEP17 <2.0; FISH negative was identified when the ratio of HER2/CEP17<2.0 (24). TNBC was defined as ER-, PR-, and HER2- (25).

### MSI Detection by PCR With Two MSI Panels

MSI was measured using a Veriti DX 96-well PCR thermal cycler (Applied Biosystems, Foster City, CA, USA) for PCR assay with two panels of microsatellite markers {Promega 1.2: BAT-25, BAT-26, NR-21, NR-24, and MONO-27; National Cancer Institute (NCI): BAT25, BAT26, D2S123, D5S346, and D17S250}, respectively, and a 3500 Dx Genetic Analyzer (Applied Biosystems, Foster City, CA, USA) for PCR product detection after DNA extraction from formalin-fixed paraffin-embedded tumor tissue and paired peritumoral benign tissue. The sample was considered to be microsatellite unstable if there was a shift of three base pairs in the tumor allele compared with normal tissue. MSI-H, MSI-L (low-frequency microsatellite instability), and MSS were distinguished when two or more, one, and no unstable markers were observed, respectively (26).

### PD-L1, LAG-3, and CD8 Protein Expression Testing by IHC

IHC staining of three antibodies, including PD-L1 (clone E1L3N, dilution 1:200; Cell Signaling Technology, MA, USA), LAG-3 (clone D2G40, dilution 1:150; Cell Signaling Technology, MA, USA), and CD8 (clone 4B11, Leica, Newcastle upon Tyne, UK) were carried out using the DAKO EnVision method on 4 μm sections according to the manufacturers’ protocols, respectively. Positive PD-L1 expression was interpreted when there was membranous staining with or without cytoplasmic staining of any intensity in ≥1% of tumor cells or immune cells as described previously (12). LAG-3 and CD8 were respectively defined as positive when there were intra tumoral and peri-tumor stromal lymphocytes with any immunoreactivity in ≥1% or in ≥10% of the entire tumoral area according to published studies and the recommendations of the International TILs Working Group (12, 27).

### Statistical Analysis

The data were analyzed using SAS version 9.4 software. Significance was considered at a P-value < 0.05.

## Results

### MMR and MSI Status

**Figure 1:**
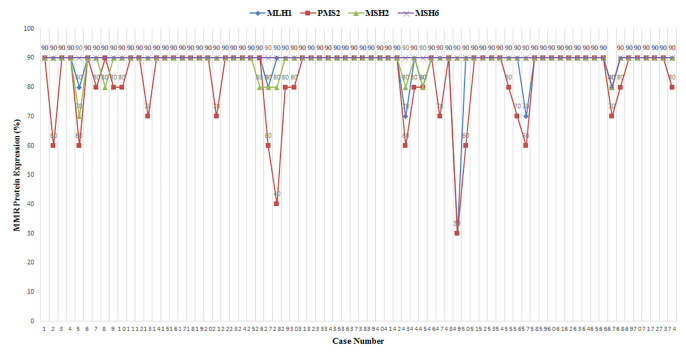
MLH1, PMS2, MSH2, and MSH6 protein expression in 74 triple-negative breast cancer (TNBC) samples.

**Figure 2:**
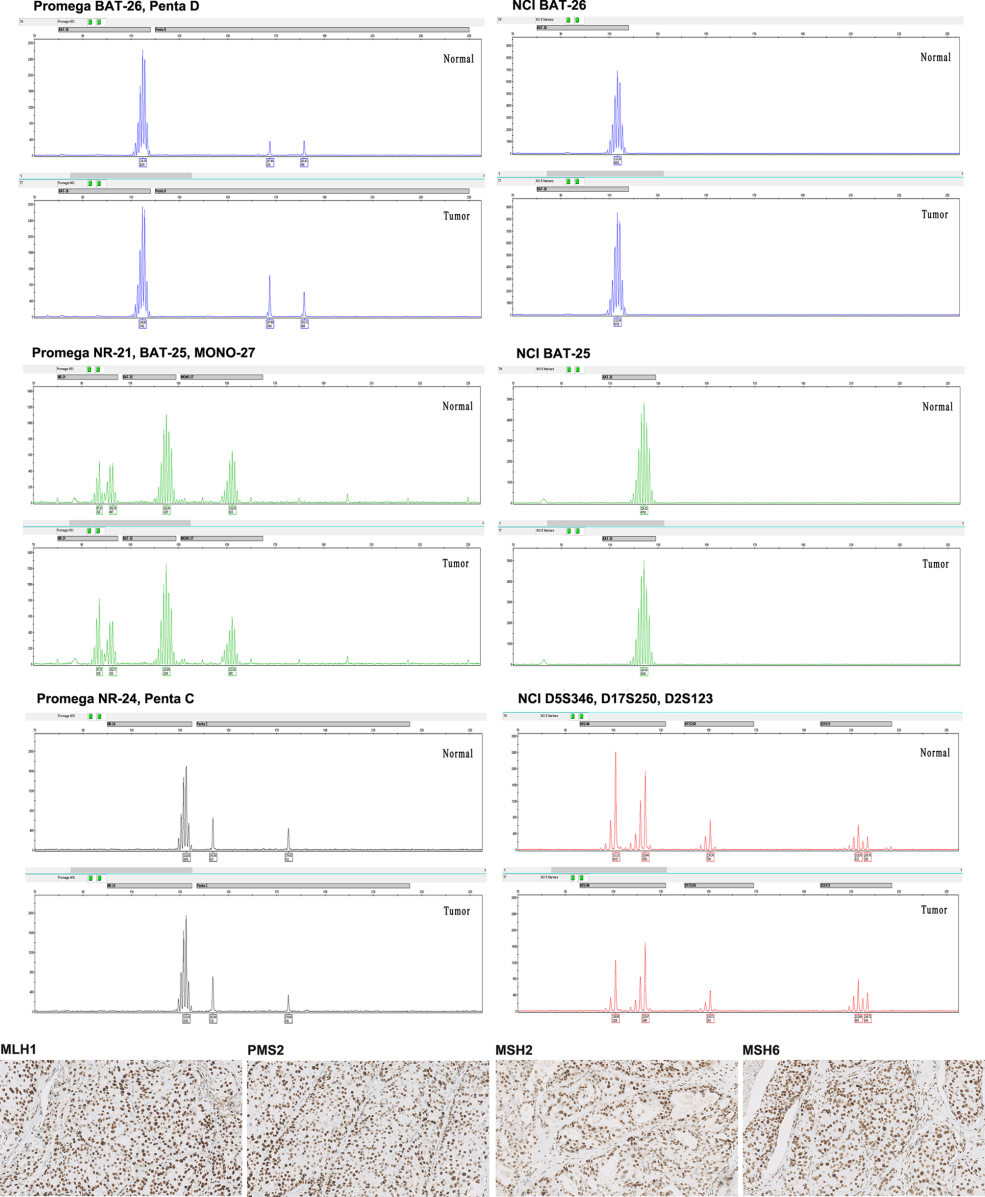
Representative images from pMMR/MSS triple-negative breast cancer (TNBC) in our cohort. The four MMR proteins (MLH1, PMS2, MSH2, and MSH6) (X200) all showed intact immunohistochemistry (IHC) staining. The microsatellite markers presented MSS both by Promega 1.2 and NCI panels. Promega 1.2 panel: BAT-25, BAT-26, NR-21, NR-24, and MONO-27; NCI panel: BAT25, BAT26, D2S123, D5S346, and D17S250.

Four MMR proteins, MLH1, MSH2, MSH6, and PMS2, were homogenously expressed in all samples; all were pMMR. No heterogeneous expression was observed in our cohort. Except for one sample with low-expressed MLH1 and two samples with low-expressed PMS2, high expression of MMR proteins in all other cases was determined (**Table 2**). MMR protein expression is listed in **Figure 1**. Tumors with dMMR were not found in the series. All samples showed MSS detected by Promega 1.2 and NCI panels (**Figure 2**). There were no cases of MSI-H or MSI-L (**Table 3**).

**Table 2:** MMR protein expression levels in the study cohort.

	MMR protein expression by IHC		
	Average (range)		n
MLH1	88.1%(30-90)	High Low	73 1
PMS2	83.4%(30-90)	High Low	72 2
MSH2	88.8%(70-90)	High Low	74 0
MSH6	89.9%(80-90)	High Low	74 0

**Table 3:** MSI status detected by Promega 1.2 and NCI panels.

		MSI status by Promega 1.2		
		MSI-H	MSI-L	MSS
	MSI-H	0	0	0
**MSI status by NCI**	MSI-L	0	0	0
	MSS	0	0	74

### PD-L1, LAG-3, and CD8 Expression

In 43 of the 74 cases (58.1%) PD-L1 expression was identified, including 1 case (1.4%, 1/74) with tumor PD-L1+, 25 cases (33.8%, 25/74) with TIL PD-L1+, and 17 cases (23.0%, 17/74) with tumor and TIL co-expression of PD-L1, respectively. The rate of PD-L1+ TILs was remarkably higher than that of PD-L1+ tumors (P<0.001).

From the perspective of expression level, 18 cases (24.3%, 18/74) with tumor PD-L1+ (the proportion of positive cells was 1%–80%) were observed, including 16 cases (88.9%, 16/18) of low-level expression (≥1% and <50%) and two cases (11.1%, 2/18) of high-level expression (≥50%). In another subtype, 42 cases (56.8%, 42/74) with TIL PD-L1+ were determined (the proportion of positive cells was also 1%–80%), including four cases (9.5%, 4/42) of low-level expression (1%) and 18 cases (42.9%, 18/42) of high-level expression (≥50%). In summary, PD-L1 was predominantly expressed in immune cells, most of which showed high-level expression.

We recognized 20 cases with LAG-3 expression (27.0%, 20/74) with a 1%–30% proportion of positive lymphocytes, including seven cases (35.0%, 7/20) of high-level expression (≥10%). The LAG-3 positive samples were PD-L1+ (the frequency of PD-L1 and LAG-3 co-expression was 27.0%, 20/74), which accounted for 46.5% (20/43) of all PD-L1+ cases. In the LAG-3+ subtype, 10 cases (50%, 10/20) had TIL PD-L1+, nine cases (45%, 9/20) showed concurrence in tumor and immune cells for PD-L1 expression, and 1 case (5.0%, 1/20) showed tumor PD-L1+ only. In the LAG-3+ subgroup, TIL PD-L1 expression was also dramatically higher than tumor PD-L1 expression. The high correlation between TIL PD-L1 expression and LAG-3 expression was explored (P<0.01). In brief, all LAG-3+ cases expressed PD-L1 simultaneously. Most samples with concurrence of PD-L1+ and LAG-3+ were of TILs PD-L1+ or concurrence of TILs and tumor PD-L1+.

**Figure 3:**
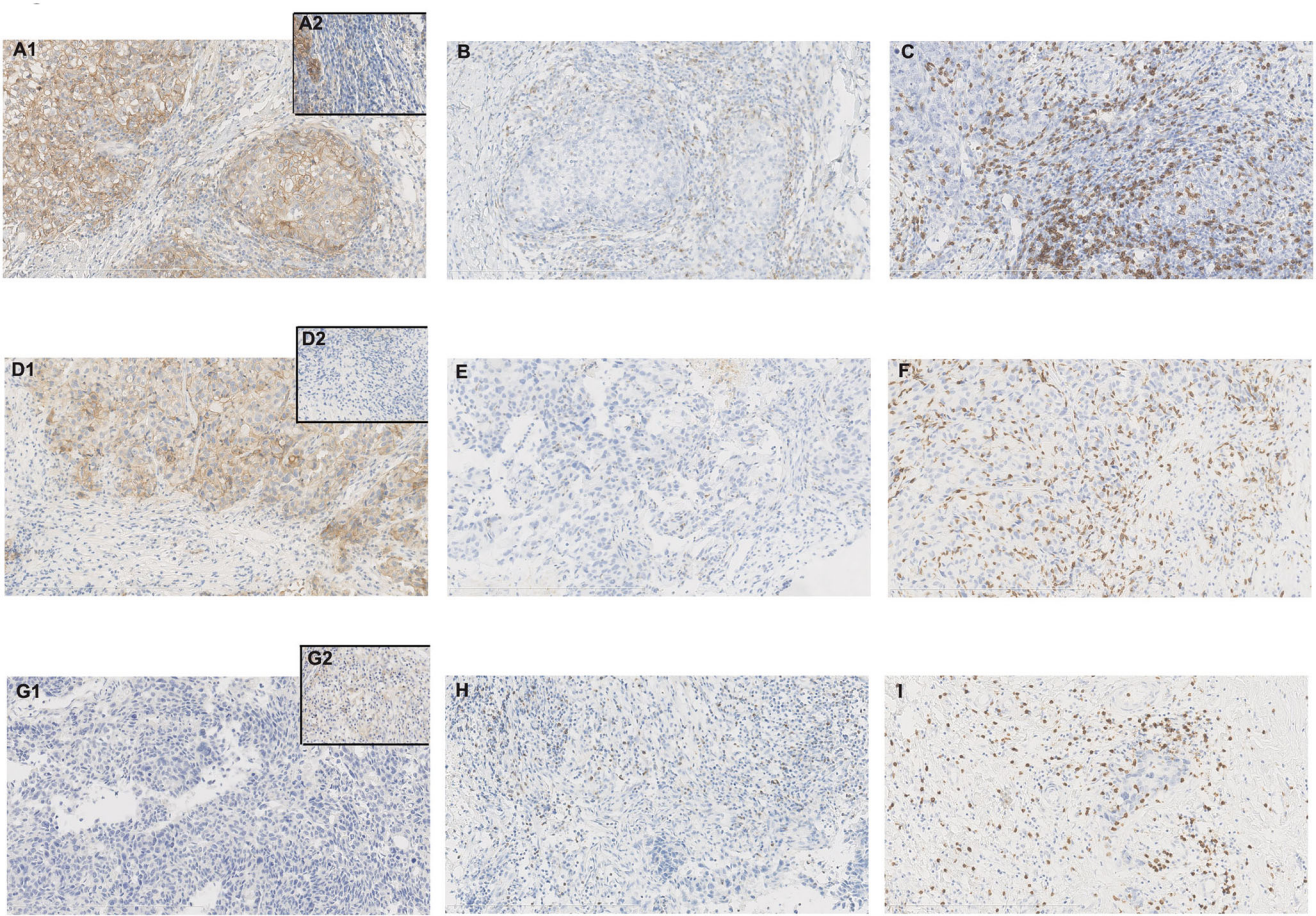
Case no. 74 **(A–C)** showed tumor programmed death-ligand 1 (PD-L1)+, tumor-infiltrating lymphocytes (TILs) PD-L1+, lymphocyte-activation gene 3 (LAG-3)+, and CD8+ with 80%, 10%, 30%, and 70% positive cells, respectively (X200). **(A)** PD-L1 staining with clone E1L3N (A1. tumor PD-L1+/partial TILs PD-L1+; A2. TILs PD-L1+); **(B)** LAG-3 staining with clone D2G40; **(C)** CD8 staining with clone 4B11; Case no. 46 **(D–F)** showed tumor PD-L1+, TILs PD-L1-, LAG-3+, and CD8+ with 80%, 0%, 1%, and 90% positive cells respectively (X200). **(D)** PD-L1 staining with clone E1L3N (D1. tumor PD-L1+/TILs PD-L1-; D2. TILs PD-L1-); **(E)** LAG-3 staining with clone D2G40; **(F)** CD8 staining with clone 4B11; Case no. 34 **(G–I)** showed tumor PD-L1-, TILs PD-L1+, LAG-3+, and CD8+ with 0%, 10%, 10%, and 70% positive cells respectively (X200). **(G)** PD-L1 staining with clone E1L3N (G1. tumor PD-L1-; G2. TILs PD-L1+); **(H)** LAG-3 staining with clone D2G40; **(I)** CD8 staining with clone 4B11.

Apart from one CD8- case (also PD-L1- and LAG-3-), high-frequency CD8 + was exhibited (98.6%, 73/74) with 20-90% positive cell proportion, including 64 cases (86.5%, 64/74) moderate or more level (≥50%) of expression, and 14 cases (18.9%, 14/74) of high-level expression (≥90%). CD8+ with high-level expression was a common feature in our patients (**Figure 3**).

**Figure 4:**
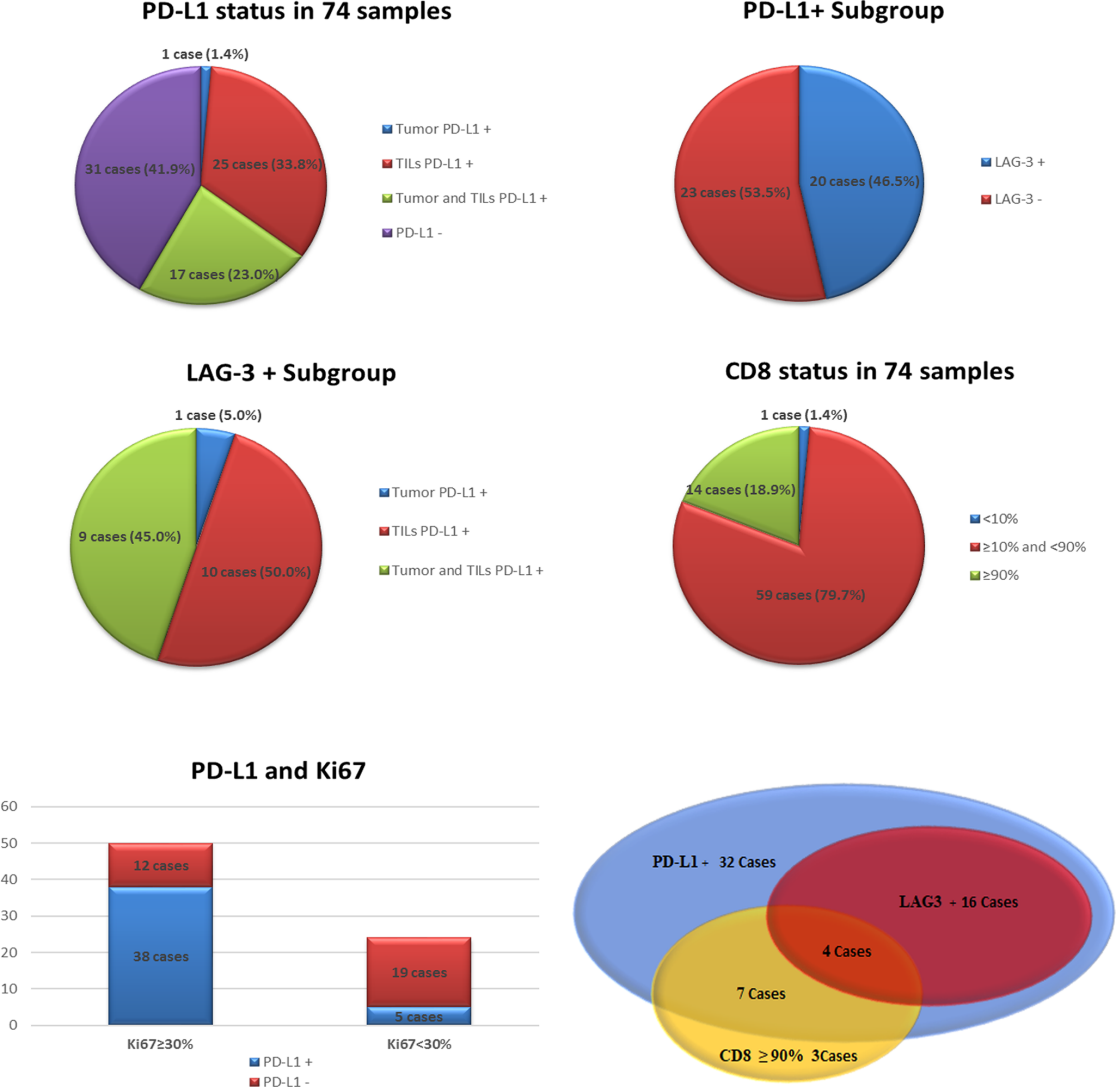
The status of programmed death-ligand 1 (PD-L1), lymphocyte-activation gene 3 (LAG-3), CD8 and Ki67 index in our series.

In addition, these samples possessed 5%–90% Ki67 index, including 50 (67.6%, 50/74) cases with high proliferation index (Ki67≥30%). The proportion of high Ki67 index in the PD-L1+ and PD-L1- subgroups was 88.4% (38/43) and 38.7% (12/31), respectively. In the subgroup of concurrent PD-L1+ and LAG-3+, 18 cases had high Ki67 index (90.0%, 18/20), except for two cases with low expression of Ki67 (5% and 25%) (**Figure 4**).

## Discussion

With the increasing application of immune components in solid tumors, the detection of potential TNBC patients who could benefit by receiving ICIs warrants further research. Therefore, it is important to explore the incidence of TNBC with dMMR/MSI-H features, which is a predictive marker approved by the FDA for solid tumors treated with ICIs (pembrolizumab), and it is also vital to identify TNBC with dMMR/MSI-H or for drafting some recommendations of immune biomarkers in the future, although this indication of ICIs is not used in China to date.

In our series, all cases were pMMR, and no dMMR samples were found. The IHC results were confirmed by both Promega 1.2 and NCI panels, and MSS status were disclosed for all samples. IHC and PCR showed high consistency. Our findings corroborated the reports that dMMR and/or MSI were rare events (<1.0%) in TNBC (13, 28). Recently, MMR gene variation in 963 cases of invasive breast cancer in TCGA (The Cancer Genome Atlas) was evaluated by a research team. They confirmed a low incidence of MMR deficiency, reporting that 2.9% of specimens harbored any mutation in at least one of the MMR genes (MLH1, PMS2, MSH2, and MSH6) as well as a low frequency of driver mutation as compared to colorectal cancer (29). In our pMMR subgroup, majority of cases with highly expressed MMR proteins were found. Only one case with low-expressed MLH1 and two cases with low-expressed PMS2 were exposed. In a previous study, dMMR was observed in 0.4% (1/285) of breast cancer cases, which were TNBC cases with loss of MLH1 and PMS2 proteins (29). In another recent study including 63 TIL-high TNBC cases from Japan, MSS was identified in all samples, and only one dMMR case with loss of MLH1 and PMS2 was reported (22). Low or loss of MLH1 and PMS2 protein expression might often occur in TNBC patients based on our results and the published data available. Low-expressed PMS2 protein was also observed in one out of 10 cases of colorectal cancer in our previous study cohort, and other MMR proteins were all highly expressed in all samples (data not shown) (26). The potential biological implications of this process remain to be explored further. We used the IHC detection system approved by the FDA for Lynch syndrome test (https://www.fda.gov/medical-devices/vitro-diagnostics/nucleic-acid-based-tests) and two accepted conventional MSI panels (Promega 1.2 and NCI) to reduce approach bias. Similar to the findings from the literature mentioned above, we did not find a high discordance between MMR protein expression by IHC and MSI status by DNA testing (NCI panel), which has been reported previously (14). Although, a study declared that the hormone receptor-positive BC possessed a similar rate of dMMR as TNBC patients (17% vs. 20%), which was also noted, response rates from PD-1 inhibitors (e.g., avelumab, an anti-PD-L1 antibody) was obviously lower than that of TNBC (14, 30). Hence, screening of TNBC patients who benefit from ICIs has been brought into focus relatively so far.

Our data showed homogeneous pMMR staining and consistent results between IHC and PCR for MMR and MSI measurement, respectively, suggesting that the two methods could be used interchangeably in TNBC notwithstanding no infrequent dMMR/MSI-H or MSI-L cases in our cohort for conclusively verifying our view. Our results of IHC (whole slide staining) were different from a tissue microarray (TMA) cohort study in which 6.9% of TNBC cases with complete MMR loss were presented (12). These differences between the two studies were probably caused by different IHC antibody clones and sample types. Two out of 228 cases (0.9%) were found to harbor MSI-H in TNBC *via* the same analysis system (Promega 1.2) as ours, which was reported by another research team. IHC was supported for MMR assessment by some researchers despite being unchecked by MSI assay (28, 31). Consequently, adequate experience of detecting MMR/MSI in TNBC is still required. Despite PD-1(L1) ICIs (such as pembrolizumab) targeting dMMR/MSI-H tumors, the beneficiary was not preselected using this immune biomarker because of the rare event in TNBC. The exploration should be focused on other effective-related biomarkers of ICIs.

Currently, PD-L1 expression is considered to be one of the most important markers for predicting ICI effect. The data from clinical settings remain limited because ICIs (such as atezolizumab) are not currently used as first-line therapy for TNBC in China. A study involving 228 cases mentioned above showed that 39.5% of Japanese TNBC expressed tumor PD-L1 (same E1L3N and cutoff values as in our study), but immune cell PD-L1 status was not evaluated (28). Moreover, a Chinese team demonstrated that PD-L1+ (using E1L3N with 5% cutoff) accounted for 25.74% and 30.79% in tumor cells and lymphocytes, respectively, in primary TNBC (32). We identified 58.1% of cases with PD-L1+, including only 1 tumor PD-L1 expression (1.4%, 1/74), which was much lower than the positive rate in lymphocytes (33.8%, 25/74). This tendency was similar to another study on 119 cases of TNBC that reported 64.4% of TILs and 0% of tumor cell PD-L1+. Accordingly, they revealed that TNBC had a higher PD-L1 expression rate than HER2+ BC (75.2% vs16.8%), which validated the findings published previously (12, 33). In addition to PD-L1 commonly expressed on immune cells, in which PD-L1 expression (≥ 1%,with any intensity) was determined as a sensitive marker for evaluating TNBC response to ICI (Impassion 130 study) (34). Our data supported TNBC patients as a potential population who benefited from ICIs and indicated the need to focus on PD-L1 status in immune cells.

Available evidence indicates that the level of TILs, which are also important biomarkers for immunotherapy in TNBC, was much higher than in other subtypes, among which cytotoxic CD8+ lymphocytes were considered as independent markers of favorable prognosis in TNBC (35, 36). Vihervuori et al. reported that when the cutoff was ≥10%, ≥50%, and ≥90%, the CD8+ rates in the tumor center and invasive front of the tumors were 54% vs. 53.5%, 8.2% vs. 8.8%, and 0 vs. 0, respectively (37). A meta-analysis demonstrated that a high number of TILs would predict prolonged overall survival (OS) regardless of TIL location (intratumoral or stromal), total TILs, or CD8+ TILs (38). Thus, in the current study, we scored fashionable CD8+ immune cells, including intratumoral and stromal infiltrating lymphocytes. The frequency of CD8+ (≥10%) T cells was up to 98.7%, among which the samples with CD8+ cells ≥50% and ≥90% were 87.8% and 18.9%, respectively. The different findings between our study and previous studies need to be further analyzed.

However, the response rates from ICIs (especially monotherapy) are usually lower because the tumor microenvironment is quite heterogeneous and have complicated interactions with biological factors that are less known. Several inhibitory checkpoints have been recognized and are being tested as promising new targets for cancer immunotherapy in addition to PD-1 (L1) blockade, including LAG-3, TIM-3 (T cell immunoglobulin and mucin domain-containing molecule-3), and TIGIT (T cell immunoreceptor with immunoglobulin and immunoreceptor tyrosine-based inhibitory motif domain) are highly anticipated (39). LAG-3 is considered the paramount target next to PD-1. At least 13 anti-LAG-3 reagents have been developed to date (40).

LAG-3 was found to be upregulated in some epithelial cancers. In addition, LAG-3 and PD-L1 showed synergism in T-cell action regulation causing immune resistance (17, 41). Inhibition of LAG-3/MHCII interaction with targeted reagents (such as IMP321) was found to activate tumor-related CD8 expression and produce cytokines (42–44). Furthermore, overexpression of LAG-3 was inferred to be one of the causes of poor response to PD-1(L1) ICIs in cancers. According to the reports, the clinical benefit of combining anti-LAG-3 (relatlimab) and anti-PD-1 (nivolumab) was observed for melanoma patients with progressive disease during prior nivolumab monotherapy, and the objective response rate (ORR) was 3 fold higher in patients with LAG-3 positive than in LAG-3 negative patients (45, 46). Semblance of LAG-3+ TILs may be a predictor of existing cancer–immune interaction and present an inflamed tumor, which indicates a better prognosis. In a phase I/II study (NCT02460224), LAG525 (an anti-LAG-3 reagent) plus spartalizumab (an anti-PD-1 reagent) showed a durable response in solid tumors including TNBC (47). In a BC study with TMA samples, 53% of PD-L1+ cases expressed LAG-3, and the proportion of concurrent LAG-3+ and CD8+, and PD-L1+ and CD8+, were 26% and 18%, respectively (data on TNBC were not available). In addition, compared with other subtypes, basal-like BC possessed more LAG-3+ cases (33%). They suggested that this may be significant for evaluating ICI anti-tumor activity in relevant clinical trials *via* stratification of PD-L1 + and double-positive PD-L1 and LAG-3 (16). In our specimens, we recognized 27.0% of LAG-3+ cases in TNBC, meaning that 46.5% of cases had concurrent PD-L1 and LAG-3 expression and high Ki67 index for most cases in the PD-L1+ subgroup. Our findings were consistent with those reported previously for BC. In summary, studies on the biological and clinical significance of LAG-3 in TNBC are extremely limited.

As mentioned above, like LAG-3, upregulation of TIM-3 or TIGIT is also associated with an immune resistance mechanism (39). Relevant data from a large cohort study strongly supported TIM-3 as a prospective target for BC immunotherapy based on their finding that 28% basal-like breast cancers and 18% non-basal TNBC possessed TIM-3 expression in intra-epithelial TILs (iTILs), respectively, and TIM-3 + iTILs significantly correlated with PD-1, LAG-3, and PD-L1 expression in BC (48). Several anti-TIM-3 agents are currently being used in clinical trials. The preliminary data showed 20% tumor regression from a phase 1 study of LY3321367 (an anti-TIM-3 antibody) monotherapy or in combination with LY3300054 (an anti-PD-L1 antibody) (49). TIGIT is another promising immune therapeutic target. Blocking TIGIT or its ligand poliovirus receptor leading to enhanced anti-tumor effects was observed in HER2 positive BC and TNBC cell lines (50). In a study of 10 fresh tumor samples from untreated TNBC patients, TIGIT overexpression was found in CD8+ and CD4+ TILs, and highly expressed TIGIT and its ligands (CD155 and CD112) were discovered in tumor cells and antigen-presenting cells (51). These data indicate that anti-TIGIT is a potentially valuable therapeutic approach for BC treatment. In a first-line therapy for non-small cell lung cancer, atezolizumab plus tiragolumab (an anti-TIGIT antibody) showed superior clinical efficacy as compared with anti-PD-L1 therapy alone recently (52). Therefore, dual PD-1(L1) and TIGIT blockade might be a promising option. However, TIM-3 and TIGIT targeting are still early in clinical research, and few reports of the therapeutic efficacy of anti-TIGIT or anti-TIM-3 including combinatorial therapies (TIGIT ICI or TIM-3 ICI plus PD-1(L1) ICI) are available in breast cancer to date. Furthermore, detailed mechanisms of anti-tumor immunotherapy, including blocking PD-1(L1), LAG-3, TIM-3, and TIGIT, are still unclear and require further research.

In conclusion, we retrospectively analyzed MMR, MSI, PD-L1, and LAG-3 status in TNBC. None of the cases demonstrated dMMR or MSI, as detected by authentic IHC assay and MSI panels, respectively. This indicates that potential beneficiaries of PD-1(L1) ICIs may not be preselected by these markers. All cases enrolled in the current study exhibited a high frequency of PD-L1+ and CD8+. Compared to tumors, PD-L1 expression in lymphocytes was more common and more attractive to investigators. Furthermore, in the PD-L1+ population, approximately half of the samples had PD-L1+ and LAG-3 co-expression, which symbolized the synergism of PD-L1 and LAG-3 in TNBC. For patients with poor responsiveness to PD-1(L1) mono immunotherapy, the possibility of benefiting from dual-blockading PD-1 and LAG-3 may not be neglected. It is worthwhile to further understand the significance of LAG-3 in TNBC.

## Data Availability Statement

The original contributions presented in the study are included in the article/supplementary materials. Further inquiries can be directed to the corresponding author.

## Ethics Statement

The studies involving human participants were reviewed and approved by The Institutional Review Board (IRB) of Peking Union Medical College Hospital (PUMCH). The ethics committee waived the requirement of written informed consent for participation.

## Author Contributions

SW, YYL, and YFL performed the experiments. SW, YYL, XW, and FM collected the clinicalpathological information. XS, JW, and SW scored the slides and analyzed the data. SW and XZ designed the study and wrote the manuscript. All authors contributed to the article and approved the submitted version.

## Funding

This study was supported by the foundation from the National Key Research and Development Program of China (No.2017YFC1309004) and Chinese Academy of Medical Sciences (CAMS) Initiative for Innovative Medicine (No.2017-I2M-1-005).

## Conflict of Interest

The authors declare that the research was conducted in the absence of any commercial or financial relationships that could be construed as a potential conflict of interest.
